# The Endogenous Hallucinogen and Trace Amine N,N-Dimethyltryptamine (DMT) Displays Potent Protective Effects against Hypoxia via Sigma-1 Receptor Activation in Human Primary iPSC-Derived Cortical Neurons and Microglia-Like Immune Cells

**DOI:** 10.3389/fnins.2016.00423

**Published:** 2016-09-14

**Authors:** Attila Szabo, Attila Kovacs, Jordi Riba, Srdjan Djurovic, Eva Rajnavolgyi, Ede Frecska

**Affiliations:** ^1^NORMENT, KG Jebsen Centre for Psychosis Research, Institute of Clinical Medicine, University of OsloOslo, Norway; ^2^Division of Mental Health and Addiction, Oslo University HospitalOslo, Norway; ^3^Department of Immunology, Faculty of Medicine, University of DebrecenDebrecen, Hungary; ^4^Department of Psychiatry, Faculty of Medicine, University of DebrecenDebrecen, Hungary; ^5^Human Neuropsychopharmacology Research Group, Sant Pau Institute of Biomedical ResearchBarcelona, Spain; ^6^Centro de Investigación Biomédica en Red de Salud MentalBarcelona, Spain; ^7^NORMENT, KG Jebsen Centre for Psychosis Research, Department of Clinical Science, University of BergenBergen, Norway; ^8^Department of Medical Genetics, Oslo University HospitalOslo, Norway

**Keywords:** dimethyltryptamine, hypoxia, sigma-1 receptor, cellular survival, cellular stress

## Abstract

N,N-dimethyltryptamine (DMT) is a potent endogenous hallucinogen present in the brain of humans and other mammals. Despite extensive research, its physiological role remains largely unknown. Recently, DMT has been found to activate the sigma-1 receptor (Sig-1R), an intracellular chaperone fulfilling an interface role between the endoplasmic reticulum (ER) and mitochondria. It ensures the correct transmission of ER stress into the nucleus resulting in the enhanced production of antistress and antioxidant proteins. Due to this function, the activation of Sig-1R can mitigate the outcome of hypoxia or oxidative stress. In this paper, we aimed to test the hypothesis that DMT plays a neuroprotective role in the brain by activating the Sig-1R. We tested whether DMT can mitigate hypoxic stress in *in vitro* cultured human cortical neurons (derived from induced pluripotent stem cells, iPSCs), monocyte-derived macrophages (moMACs), and dendritic cells (moDCs). Results showed that DMT robustly increases the survival of these cell types in severe hypoxia (0.5% O_2_) through the Sig-1R. Furthermore, this phenomenon is associated with the decreased expression and function of the alpha subunit of the hypoxia-inducible factor 1 (HIF-1) suggesting that DMT-mediated Sig-1R activation may alleviate hypoxia-induced cellular stress and increase survival in a HIF-1-independent manner. Our results reveal a novel and important role of DMT in human cellular physiology. We postulate that this compound may be endogenously generated in situations of stress, ameliorating the adverse effects of hypoxic/ischemic insult to the brain.

## Introduction

Originally thought to be an opioid receptor, the sigma-1 receptor (Sig-1R) is now classified as member of an orphan family after Su and colleagues had characterized the structural and biochemical features of the ligand binding site and identified the class as a non-opioid one (Su, 1982; Su et al., 1988). Later, based on their pharmacological characteristics and tissue expression, the distinction of Sig-1R and Sig-2R subtypes was proposed (Hellewell et al., 1994). Previous studies showed that Sig-1R is expressed not only in different regions of the brain but also in immune cells (Su, 1982; Wolfe et al., 1988), and organs like liver, kidney, and gut (Hellewell et al., 1994; Theodorou et al., 2002). Sig-1R is chiefly an endoplasmic reticulum (ER) protein located on the mitochondria-associated endoplasmic reticulum membrane (MAM) where its main role is to regulate ATP synthesis through the regulation of Ca^2+^ signaling by primarily acting as a molecular chaperone (Hayashi and Su, 2007; Su et al., 2016). Another MAM-related role of Sig-1R is to facilitate stress signaling from the ER to the nucleus through chaperoning the inositol requiring enzyme 1 (IRE1) and thereby increasing the intracellular levels of antistress and antioxidant proteins (Mori et al., 2013). Upon cellular stress, including hypoxia or oxidative stress, Sig-1R interacts with numerous receptors, ion channels, kinases, and various master regulator proteins residing on the ER, MAM, nucleus, or even in the cytosol to mobilize and fine-tune antistress responses.

Based on these complex intracellular actions the Sig-1R has been conceptualized as a “pluripotent modulator” in living systems, as a controller of cell survival and differentiation (Hayashi and Su, 2007; Mori et al., 2013) which may be involved in many human diseases (Su et al., 2016). Indeed, in the last two decades a considerable amount of clinical data demonstrated the involvement of Sig-1R in various pathologies including cancer, pain, addiction, stroke, ischemic heart disease, and many neuropsychiatric disorders (Maurice and Su, 2009). It has been reported that Sig-1R regulates a plethora of different physiological processes predominantly associated with cellular differentiation, survival (Hayashi and Su, 2007; Mori et al., 2013), and immunity (Szabo et al., 2014). Recent *in vitro* and *in vivo* reports suggest that Sig-1R agonists possess potent protective effects in hypoxia and neurotoxicity models (Katnik et al., 2006; Klouz et al., 2008; Penas et al., 2011; Mancuso et al., 2012). Sig-1R and Sig-2R both have been found to modulate neuronal and microglial responses to ischemia (Cuevas et al., 2011a,b), and specific Sig-1R stimulation was shown to protect against the formation of ischemic lesions subsequent to stroke (Ruscher et al., 2012).

In mammals, the endogenous ligands of Sig-1R include neurosteroids (e.g., pregnenolone, dehydroepiandrosterone, progesterone, etc., Baulieu, 1998), and naturally occurring tryptamines such as N,N-dimethyltryptamine (DMT; Fontanilla et al., 2009). In early studies, DMT was shown to be present in various animal tissues and now is considered to be an endogenous trace amine neurotransmitter that regulates several physiological functions including neural signaling and brain/peripheral immunological processes through the Sig-1R (Su et al., 2009; Shen et al., 2010; Frecska et al., 2013; Szabo et al., 2014). In addition to its centuries-long use as a sacramental medicine within the circles of South American natives (e.g*., yopo, ayahuasca, yagé*), DMT was shown to be synthesized in the mammalian lung (Axelrod, 1961) and brain (Saavedra and Axelrod, 1972) and was found in human blood, urine, and cerebrospinal fluid (Franzen and Gross, 1965; Beaton and Morris, 1984; McKenna and Riba, 2015). Furthermore, evidence suggests that DMT can be sequestered into and stored in the vesicle system of the brain and environmental stress increases its CNS levels in mammals (Barker et al., 1981; Fontanilla et al., 2009). However, the exact role of DMT in mammalian physiology is yet to be understood.

Hypoxia induces immense alterations in the phenotype and function of cells by provoking increased expression of numerous genes. One of these major changes include the hypoxic upregulation of the hypoxia-inducible factor (HIF)-1 which consists of an α subunit (HIF-1α) and a constitutively expressed β subunit. The presence of oxygen causes the immediate cytoplasmic degradation of HIF-1α, while in hypoxia the rapid accumulation of HIF-1α and its subsequent association with the β subunit leads to the formation of an active transcription factor that translocates to the nucleus and binds to the promoters of oxygen-sensitive genes, such as the vascular endothelial growth factor (VEGF). Thus, HIF-1α is widely considered as a cellular indicator of hypoxic stress or state (Semenza, 2002).

The application of human induced pluripotent stem cells (iPSCs)/neural stem cells (NSCs) in order to elucidate the cellular and molecular details of neurological and psychiatric disorders has become an increasing trend in modern science. The lack of appropriate animal models and the unavailability of human brain tissue pose a significant drawback in biomedical investigations. Thus, *in vitro* iPSC-derived neurons are emerging as promising models both in single-cell and in simple network-based neurobiological research (Heilker et al., 2014).

Monocyte-derived macrophages (moMACs) and dendritic cells (moDCs) are critical players of immune defense in higher vertebrates. They are present in virtually all tissues of the body and, by continuously sampling their environment for self- and non-self-ligands, maintain immunosurveillance and control tissue protection and regeneration (Steinman and Banchereau, 2007; Szabo and Rajnavolgyi, 2013). *In vitro* differentiated moMACs and moDCs are frequently used in different clinical and experimental settings (Cheong et al., 2010; London et al., 2013). Furthermore, microglial and moMAC populations of the CNS are comparable concerning their phenotypic and functional properties (London et al., 2013) and are considered as gold standards in immunology and regularly used in various clinical and experimental settings (Cheong et al., 2010; London et al., 2013). They have been suggested as comparable with the microglial populations of the brain and thus may be considered as microglia-like cells (London et al., 2013). Very recently, human monocytes have been reported to migrate to the brain and are able to modulate the neuroimmune profile of the CNS (Wohleb et al., 2013). Thus, within the specific tissue setting of the brain, moMACs and moDCs may represent microglia-like cell types which—besides, in concert with, or similar to microglia—could significantly contribute to the physiological regulation of the neural tissue.

As Sig-1R activation has already been reported to be massively protective in various *in vitro* and *in vivo* ischemia and hypoxic-shock settings (Katnik et al., 2006; Klouz et al., 2008; Cuevas et al., 2011a,b; Penas et al., 2011; Mancuso et al., 2012), our goal was to test the hypothesis whether the DMT-mediated activation of Sig-1R alleviates the effects of hypoxic stress on human primary cells using iPSC, moMAC, and moDC models.

## Materials and methods

### Cell types, isolation, culturing, and phenotyping

Human iPSC-derived neural progenitor stem cells were obtained from Axol Bioscience (Little Chesterford, UK) and were differentiated to cerebral cortical neurons in approximately 35–40 days following the recommended protocol. Phenotyping of fully differentiated cortical neurons was performed by flow cytometry using anti-CUTL1 and anti-Ctip2 (both from Abcam, Cambridge, UK) and isotype-matched control antibodies (BD Biosciences, Franklin Lakes, NJ) in accordance with the recent literature (Saito et al., 2011; Sakakibara et al., 2012).

Fluorescence intensities were measured by FACS Calibur (BD Biosciences) and data were analyzed by the FlowJo software (Tree Star, Ashland, OR). Further information about the characterization of cerebral cortical neurons (phenotyping and transcriptome analysis) is available at the company website (http://www.axolbio.com/page/cortical-neurons).

Leukocyte-enriched buffy coats were obtained from healthy blood donors drawn at the Regional Blood Center of the Hungarian National Blood Transfusion Service (Debrecen, Hungary) in accordance with the written approval of the Director of the National Blood Transfusion Service and the Regional and Institutional Ethics Committee of the University of Debrecen, Faculty of Medicine (Debrecen, Hungary). Written informed consent was obtained from the donors prior blood donation and their data were processed and stored according to the directives of the European Union. Peripheral blood mononuclear cells (PBMCs) were separated by a standard density gradient centrifugation with Ficoll-Paque Plus (Amersham Biosciences, Uppsala, Sweden). Monocytes were purified from PBMCs by positive selection using immunomagnetic cell separation with anti-CD14 microbeads according to the manufacturer's instruction (Miltenyi Biotech, Bergisch Gladbach, Germany). After separation on a VarioMACS magnet, 96–99% of the cells were CD14^+^ monocytes as measured by flow cytometry. For moDC generation, monocytes were cultured in 12-well tissue culture plates at a density of 2 × 10^6^ cells/ml in AIMV medium (Invitrogen, Carlsbad, CA) supplemented with 80 ng/ml GM-CSF (Gentaur Molecular Products, Brussels, Belgium) and 100 ng/ml IL-4 (Peprotech EC, London, UK). On day 2, the same amounts of GM-CSF and IL-4 were added to the cell cultures, and moDCs were harvested on day 5. For moMAC differentiation, 50 ng/ml M-CSF (Gentaur) was added to the monocyte cultures on days 0 and 2, and fully differentiated macrophages were collected on day 4. Phenotyping of moDCs and moMACs was carried out by flow cytometry using anti-CD209-PE, anti-CD14-PE (Beckman Coulter, Hialeah, FL), anti-CD68-PE, anti-HLA-DR-FITC, and isotype-matched control antibodies (BD Biosciences).

### Induction of hypoxia in *in vitro* cell cultures

Induction of hypoxia was performed following the modified version of a previously described protocol (Wu and Yotnda, 2011). To allow culture media to de-gas, 12-well plates containing serum-free AIMV were pre-incubated in low-oxygen atmosphere (94.5% N_2_, 5% CO_2_, 0.5% O_2_) for 4 h using a hypoxia chamber (Billups-Rothenberg, San Diego, CA). Cells were then seeded on the plates, placed in the chamber and were incubated at 37°C for up to 6 h under similar hypoxia conditions. An automated regulator (with built-in flow meter and oxygen-sensor) was used to ensure and maintain the proper composition of gas mixture within the chamber. After hypoxia treatment cells were removed from the chamber and were either immediately lysed (for Western blot or QPCR), or placed on ice (for Annexin V-FITC staining and flow cytometry analysis).

### DMT treatment and sampling of cells

N,N-dimethyltryptamine (R&D Systems, Abingdon, UK) and BD1063 dihydrochloride (Tocris, Bristol, UK) were used at working concentrations of 1–200 and 1–100 μM, respectively. BD1063 treatments always preceded the addition of DMT by 30 min to allow successful antagonism. DMT, as a controlled substance (Schedule I drug), was used with the approval and monitoring of the Hungarian Institute for Forensic Sciences and the Hungarian National Police Department.

To prepare cell lysates for Western blotting and QPCR measurements, *in vitro* cell cultures were sampled after 6 h of treatment (if not stated otherwise).

### RNA isolation, cDNA synthesis, and QPCR

Real-time quantitative polymerase chain reaction (QPCR) was performed as described previously (Szabo et al., 2016). Briefly, total RNA was isolated by TRIzol reagent (Invitrogen, Carlsbad, CA). 1.5–2 μg of total RNA were reverse transcribed using SuperScript II RNase H reverse transcriptase (Invitrogen) and Oligo(dT)15 primers (Promega, Madison, WI). Gene-specific TaqMan assays (Applied Biosystems, Foster City, CA) were used to perform QPCR in a final volume of 12 μl in triplicates using AmpliTaq Gold DNA polymerase and ABI StepOnePlus real-time PCR instrument (Applied Biosystems). Amplification of 36B4 was used as a normalizing control. Cycle threshold values (Ct) were determined by using the StepOne 2.1 software. Constant threshold values were set for each gene throughout the study. The sequence of the primers and probes are available upon request.

### Western blotting

Cells were lysed in Laemmli buffer and the protein extracts were tested by Ab specific for Sig-1R/OPRS1, HIF-1α, ATF6, p65, phospho-p65 (S536) (all from Abcam), and β-actin (Sigma, Schnelldorf, Germany) diluted at 1:500 and 1:000, respectively. Anti-rabbit Ab conjugated to horseradish peroxidase (GE Healthcare, Little Chalfont Buckinghamshire, UK) was used as the secondary Ab at a dilution of 1:5000. The SuperSignal enhanced chemiluminescence system was used for probing target proteins (Thermo Scientific, Rockford, IL). After the membranes had been probed for Sig-1R/OPRS1 or HIF-1α, they were stripped and re-probed for β-actin.

### Cellular viability assays

The percentage of apoptotic cells was assessed by using an Annexin V apoptosis kit (BioVision, CA, USA) following the manufacturer's recommendations. The rate of necrotic cell death was also monitored simultaneously by measuring membrane integrity. Necrotic cell death was quantified based on the loss of membrane integrity and the uptake of propidium iodide (PI). Upon stimulation cells were harvested and stained with PI (10 μg/ml) and analyzed immediately by flow cytometry.

### RNA interference

Gene-specific siRNA knockdown was performed by Silencer Select siRNA (Applied Biosystems) transfection using Gene Pulser Xcell instrument (Bio-Rad, Hercules, CA). Pulse conditions were square-wave pulse, 500 V, 0.5 ms. Immediately after electroporation, moMACs/moDCs were transferred to pre-warmed, fresh medium supplemented with penicillin, streptomycin, and L-glutamine. Gene knockdown in neurons was performed with the LyoVec transfection system (InvivoGen, San Diego, CA) according to the manufacturer's recommendations. Silencing of Sig-1R gene expression was performed by using a mix of three of the available Sig-1R siRNAs. Silencer negative control non-targeting siRNA (Applied Biosystems) was used as a negative control. The efficacy of siRNA treatments was tested 2 days post-transfection by Western blotting.

### Statistical analysis

Data are presented as mean ± SEM. A *t-test* was used for comparison of two groups followed by Bonferroni correction. Two-way ANOVA was used for multiple comparisons. Differences were considered to be statistically significant at *p* < 0.05 (^*^).

## Results

### Differentiation-dependent expression of Sig-1R in human iPSC-derived cortical neurons

**Figure 1:**
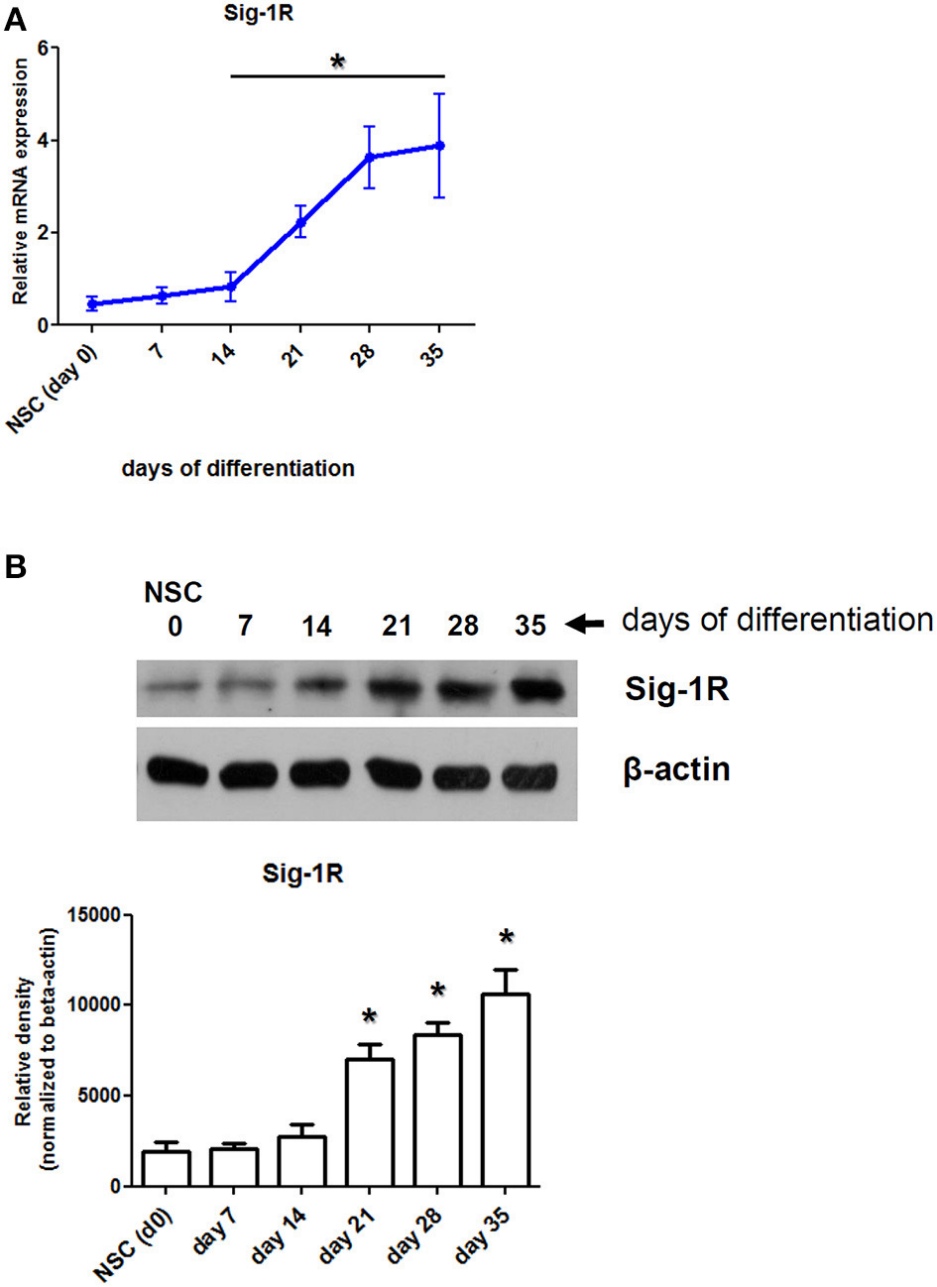
**Expression of Sig-1R in differentiating human primary iPSC-derived cortical neurons. (A)** Relative mRNA expression of Sig-1R in cortical neurons during the differentiation process from iPSC-derived neural stem cells (NSC). Results represent the Mean ± SEM of four independent experiments. **(B)** Sig-1R protein expression in differentiating cortical neurons measured by Western blot. Result of a typical experiment out of four is shown. Densitometry data show the Mean ± SEM of four independent measurements. Asterisk indicates statistical significance (*p* < 0.05).

In this work, we used three experimental models—moMACs, moDCs, and iPSC-derived neurons—to investigate the effects of DMT-mediated activation of the Sig-1R in hypoxia. In a previous study, we have already showed that human moMACs and moDCs express high levels of the Sig-1R gene and protein (Szabo et al., 2014). Though the expression of Sig-1R has also been demonstrated in human neural cell types (reviewed in Frecska et al., 2013), it has not been investigated in NSCs and in iPSC-derived cortical neurons. Therefore, we first sought for the Sig-1R expression profile of iPSC-derived neurons during the differentiation process. We found that NSCs have a low baseline expression of Sig-1R at both the mRNA and protein levels and its expression increases during the differentiation of cells into cortical neurons (Figure 1). The expression of Sig-1R becomes prominent after the 14th (mRNA) and 21st (protein) days of differentiation where significant changes were detected as compared to the baseline Sig-1R expression of NSCs (Figures 1A,B). The highest level of Sig-1R was detected at the end of the differentiation process (Figure 1).

### *In vitro* DMT-treatment of human primary cells results in increased survival in severe hypoxic environment

Based on our previous findings about the detectable levels of Sig-1R in human iPSC-derived neurons (Figure 1), moMACs, and moDCs (Szabo et al., 2014), we next investigated whether the *in vitro* treatment of these cell types with DMT, as a natural endogenous ligand of Sig-1R (Fontanilla et al., 2009), influences their survival in hypoxia. Human tissues experience a wide range of diverse oxygen tensions that profoundly differ from that of the inhaled ambient oxygen (21%, 160 mm Hg). By the time inhaled oxygen reaches tissues and organs its tension drops to 2–9% (14–65 mm Hg; Brahimi-Horn and Pouysségur, 2007). Thus, in various experimental human tissue cultures, 2–9% *in vitro* oxygen level is considered as physiologic normoxia (Simon and Keith, 2008; Mohyeldin et al., 2010). Certain low vascular density tissues may experience even lower oxygen tensions, however, 1% or lower level of oxygen is often regarded as a hypoxic environment in the literature (Semenza, 1999; Liu and Simon, 2004). In our experiments we used severe hypoxic treatment of cells where, in accordance with other studies (Lee et al., 2013; Harrison et al., 2015), the level of oxygen was set to 0.5% in a hypoxia chamber.

**Figure 2:**
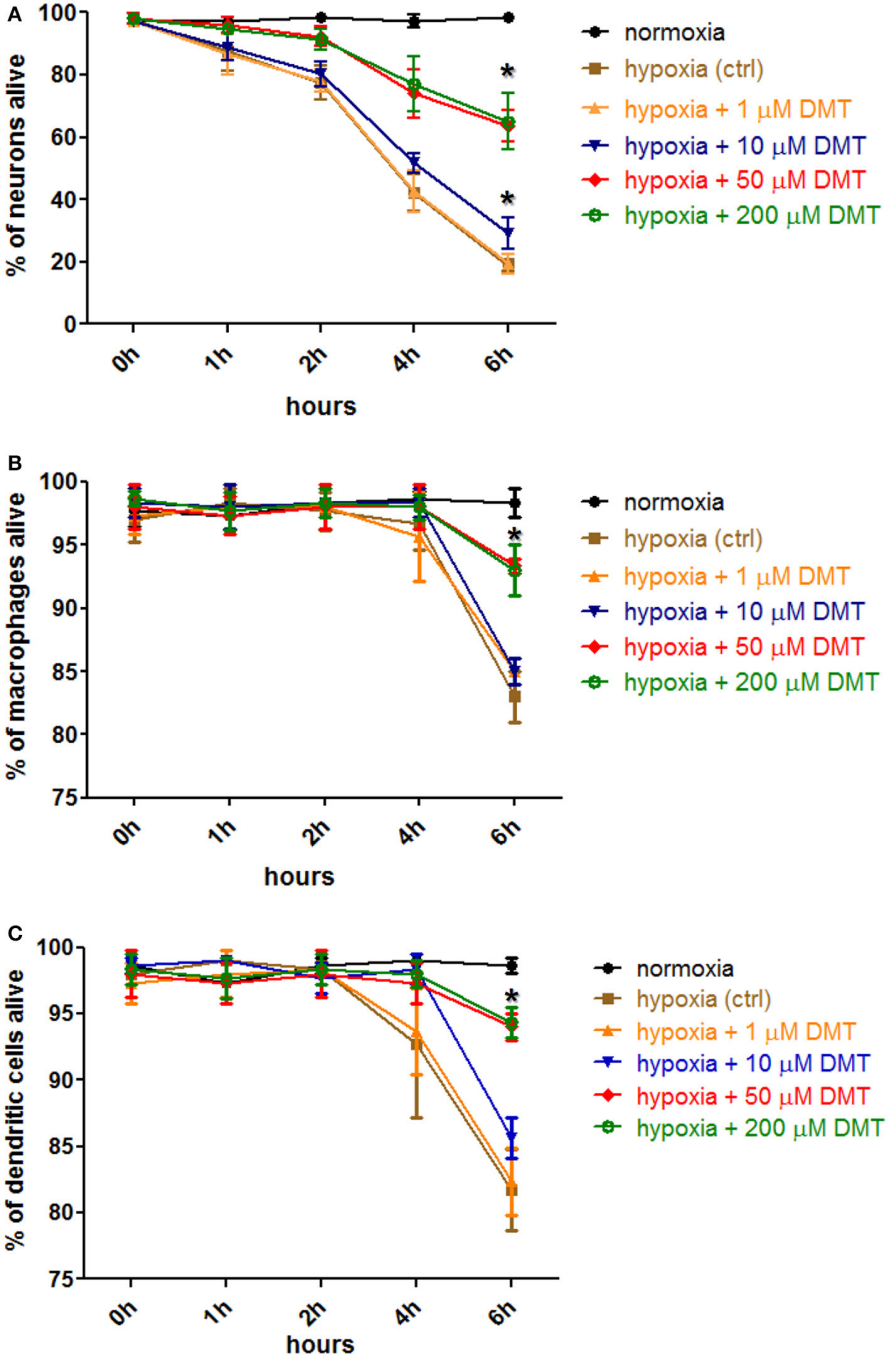
**The effect of DMT treatment on the survival of human iPSC-derived cortical neurons, monocyte-derived macrophages and dendritic cells in hypoxic environment**. Hypoxia treatment (0.5% O_2_) of *in vitro* cell cultures and cellular viability assays were carried out as described in the Section Materials and Methods. Prior to hypoxia treatment, human primary iPSC-derived cortical neurons **(A)**, moMACs **(B)**, and moDCs **(C)** were either treated with 1–200 μM DMT or left untreated (hypoxia ctrl). Normoxic cultures were used as positive controls (normoxia). Induction of cellular death was assessed by Annexin V-FITC staining. Flow cytometry data of three (neurons) or six (macrophages and dendritic cells) independent experiments are shown as Mean ± SEM. In each case, asterisk indicates significance compared to hypoxia controls (*p* < 0.05).

Incubation in hypoxic environment rapidly induced cell death in all cultures as assessed by Annexin V-FITC staining and subsequent flow cytometry analysis (Figure 2). The ratio of necrotic cells, as measured with PI-staining, never exceeded 6% median values (±1%, *n* = 3 in neuron, and ±3%, *n* = 6 in moMAC/DC cultures; data not included in Figure 2). By using different concentrations (1–200 μM) we found that *in vitro* administration of 10–200 μM DMT increases the survival of iPSC-derived cortical neurons (Figure 2A), moMACs (Figure 2B), and moDCs (Figure 2C) in hypoxia. This survival-boosting effect was statistically significant at DMT concentrations of as low as 10 μM (neurons) or 50 μM (moMAC/DCs) as compared to the non-DMT-treated hypoxia controls (Figure 2). Non-DMT-treated control cultures (hypoxia controls) of iPSC-derived neurons showed significant increase in the ratio of apoptotic cells even after 1 h of hypoxia treatment as compared to normoxic controls (Figure 2A). Six hours of hypoxia resulted in a median of 19% (±2%, *n* = 3) survival rate in case of non-treated iPSC-derived cortical neuron control cultures, while the administration of 10 and 50 μMs DMT elevated this value to a median of 31% (±6%, *p* = 0.037) and 64% (±5%, *p* = 0.006), respectively (Figure 2A). Interestingly, in cases of moMACs and moDCs significant amounts of apoptotic cells could be detected only after 6 h of hypoxia (Figures 2B,C). Furthermore, no difference was seen between the modulating effects of 50 and 200 μM concentrations of DMT (Figure 2). Normoxic cultures exhibited no sign of change in cellular viability neither in controls (Figures 2A–C) nor in DMT-treated (normoxia+1–200 μM DMT) cases (data not shown). Since 50 μM DMT, an achievable serum concentration reported by previous *in vivo* human (Strassman and Qualls, 1994) and animal studies (Shen et al., 2011), was found to be optimal for modulating the survival capacity of all cell types this concentration was used in further experiments.

### DMT modulates the expression and function of HIF-1α in human iPSC-derived neurons, monocyte-derived macrophages, and dendritic cells under hypoxia

Our results demonstrated that severe hypoxia (0.5% O_2_) induced apoptotic death of human primary cells and DMT treatment could prevent this phenomenon (Figure 2). The expression of the transcription factor HIF-1α is strongly induced by hypoxia in many cell types, however, it is subject to ubiquitination and rapid degradation under normoxia (Huang et al., 1998; Kallio et al., 1999). The molecular basis of this process is an O_2_-dependent hydroxylation of proline residues. Under hypoxia, HIF-1α is promptly assembled and carries out the downstream control of the expression of many genes related to hypoxic stress including VEGF. Thus, increased expressions of both HIF-1α and its target gene VEGF often signify hypoxic stress or state (Semenza, 2002). We next aimed to investigate whether HIF-1α was involved or affected in this process.

**Figure 3:**
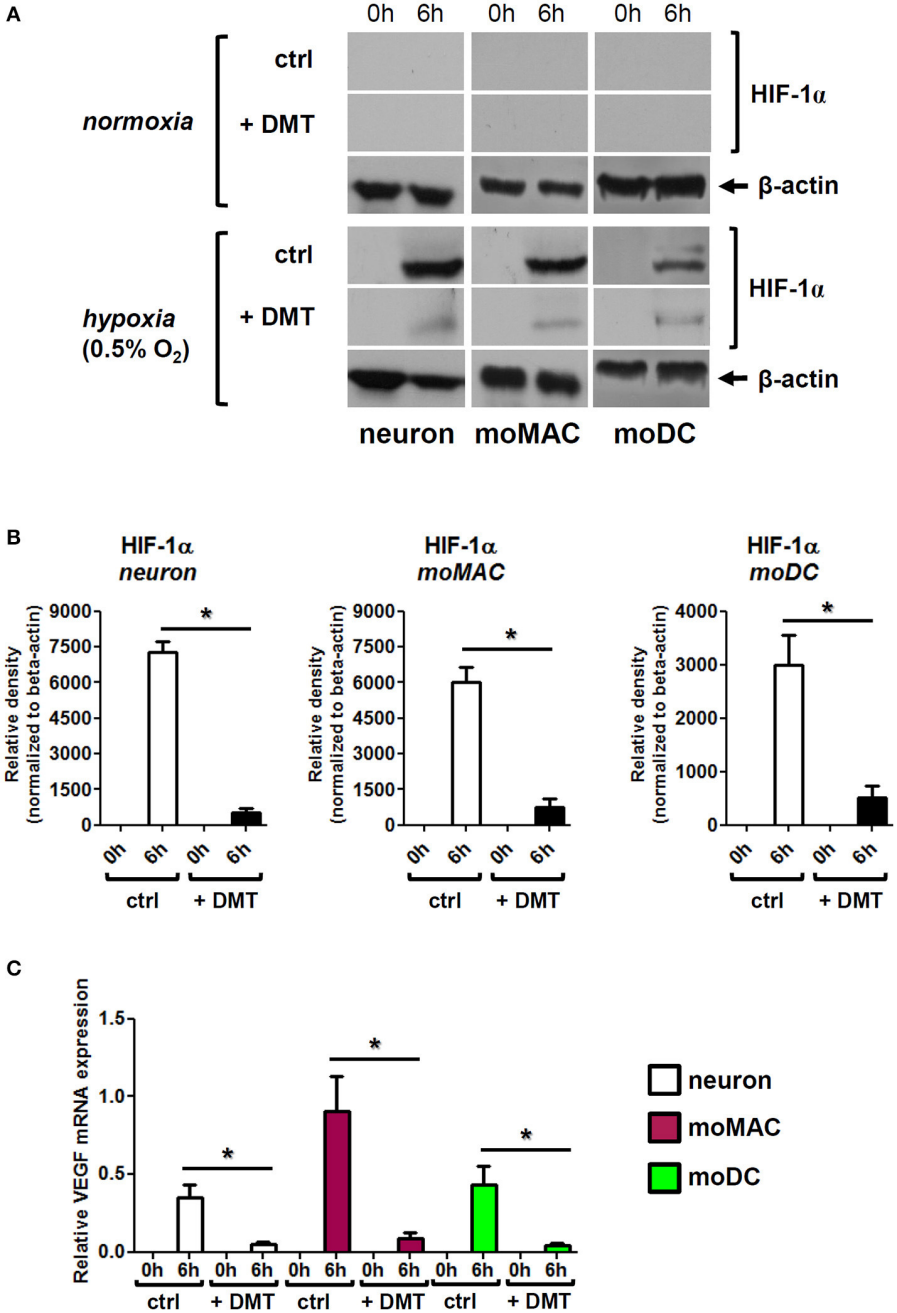
**DMT interferes with the expression and function of HIF-1α in human primary cells. (A)** Prior to their placement into hypoxia chamber (0.5% O_2_), cells were treated with 50 μM DMT (+DMT) or left non-treated (ctrl). Protein level expression of HIF-1α was evaluated by Western blotting after 6 h of hypoxia treatment (6 h) in comparison with the baseline expression (0 h). A control experiment was performed at normal oxygen level (normoxia). Results of a typical experiment out of three is shown. **(B)** Densitometry analysis of HIF-1α Western blot data in hypoxia using the same experimental setup as in panel **(A)**. Relative density values are presented as Mean ± SEM of three independent hypoxia experiments. **(C)** Relative gene expression of VEGF in human primary cells under hypoxia; treatments were performed as above panel **(A)**. Data are presented as Mean ± SEM of three independent hypoxia experiments. Asterisks indicate statistical significance (*p* < 0.05). Neuron, human iPSC-derived cortical neuron; moMAC, human monocyte-derived macrophage; moDC, human monocyte-derived dendritic cell.

We found that 6 h of hypoxia treatment greatly induced the protein-level expression of HIF-1α in human iPSC-derived neurons, moMACs, and moDCs, and the administration of 50 μM DMT prevented this increase in all cell types (Figures 3A,B). In normoxia control experiments, DMT alone did not influence HIF-1α expressions (Figure 3A). The DMT-mediated inhibition of HIF-1α protein expression under hypoxia was found to be statistically significant as compared to hypoxia controls (Figure 3B). These results were consistent with our subsequent findings showing significantly decreased mRNA expressions of VEGF upon DMT treatment in all cell types under hypoxia (Figure 3C). Furthermore, we also monitored other stress-related pathways including NF-κB and the ER-stress sensor Activating transcription factor 6 (ATF6; Elbarghati et al., 2008). We found no alterations in the protein level expression of native p65 NF-κB subunit neither could detect its phosphorylated form in our cell types in hypoxic vs. normoxic conditions within the time-range of observation (data not shown). Interestingly, the level of the 50 kDa transcriptionally active form of ATF6 showed some degree of decrease when cells were treated with DMT under hypoxia as compared to non-treated controls. However, this change did not appear to be statistically significant (Figure S1).

### Sig-1R is indispensable for the DMT-mediated modulation of cellular survival and HIF-1α expression in human iPSC-derived neurons, moMACs, and moDCs under hypoxia

In previous experiments we showed that Sig-1R is expressed in human iPSC-derived neurons (Figure 1), moMACs, and moDCs (Szabo et al., 2014). We also demonstrated that DMT treatment of these cell types can significantly increase their survival under severe hypoxia (Figure 2) and decrease their expression of HIF-1α and VEGF (Figure 3). As DMT has previously been described as a natural endogenous agonist of Sig-1R (Fontanilla et al., 2009) we next tested whether Sig-1R was involved in the observed cell biological effects of DMT.

**Figure 4:**
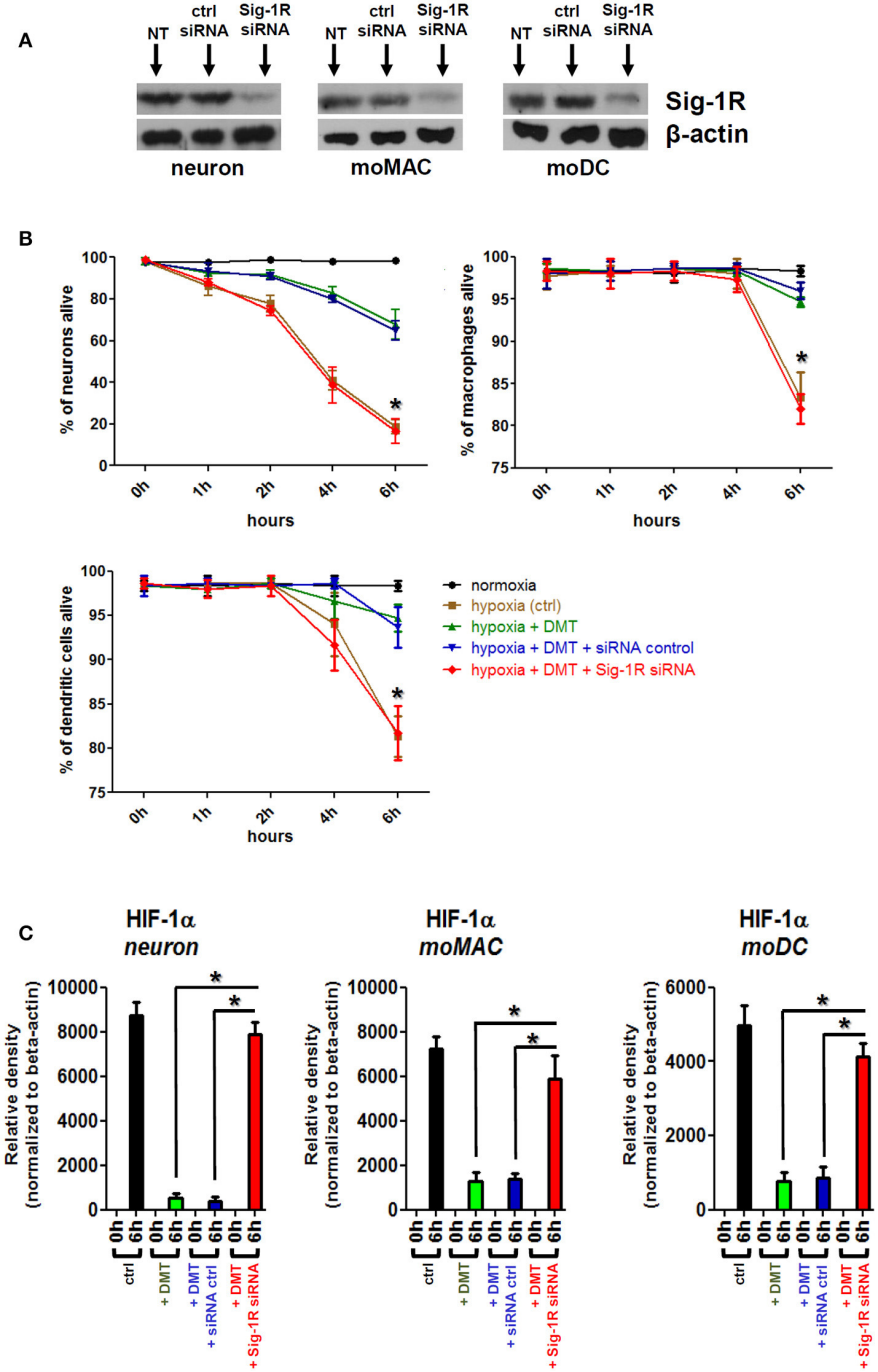
**The effect of Sig-1R gene silencing on the DMT-mediated cell survival and HIF-1α expression of human iPSC-derived cortical neurons, moMACs and moDCs in hypoxia**. Gene knockdown of Sig-1R was performed as in Section Materials and Methods. NT, non-treated control; ctrl siRNA, non-targeting negative control siRNA; Sig-1R siRNA, Sig-1R-specific siRNA. **(A)** Western blot validation of Sig-1R silencing. A typical experiment out of three (neuron) or four (moMAC/moDC) is demonstrated. **(B)** Effect of Sig-1R knockdown on cell viability in hypoxia. Cellular survival was monitored as in Figure 2. Cells were treated with 50 μM DMT before hypoxia treatment (hypoxia+DMT) or left untreated (hypoxia ctrl). Cultures with prior exposure to targeting Sig-1R siRNA (hypoxia+DMT+Sig-1R siRNA) or scrambled oligo (hypoxia+DMT+siRNA control) were also tested within the same experimental setup. **(C)** Densitometry data of HIF-1α Western blots in hypoxia following Sig-1R gene silencing. Black bars: HIF-1α protein expression after 6 h of hypoxia treatment (6 h) as compared to baseline (0 h); green bars: baseline (0 h) vs. 6 h hypoxia + 50 μM DMT treatment; blue bars: baseline (0 h) vs. hypoxia + 50 μM DMT administration in control siRNA-treated cultures; red bars: baseline (0 h) vs. 6 h hypoxia + 50 μM DMT treatment in targeting Sig-1R siRNA-treated cultures. **(B,C)** Results of three independent experiments are shown as Mean ± SEM. Asterisk indicates significance as compared to control siRNA (*p* < 0.05).

To clarify the DMT-dependent modulatory role of Sig-1R in human primary cells and to check its contribution to the observed phenomena we performed Sig-1R gene knockdown experiments. Specific silencing of the Sig-1R gene resulted in a >93% (±5%, *n* = 3) of downregulation of the Sig-1R protein in human iPSC-derived neurons as compared to non-transfected and scrambled siRNA controls, and >96% (±3%, *n* = 4) and >91% (±5%, *n* = 4) in human moMACs and moDCs, respectively (Figure 4A). Using the same treatment protocols as in Figures 2, 3, we found that specific silencing of Sig-1R abrogated the modulatory potential of DMT on the survival (Figure 4B) and HIF-1α protein expression of human iPSC-derived neurons, moMACs, and moDCs (Figure 4C). In DMT-treated Sig-1R knockdown cultures, the survival of all cell types was found to be significantly lower as compared to controls (Figure 4B). Similarly, Sig-1R gene silencing resulted in the ablation of the modulatory capacity of DMT on HIF-1α protein expression (Figure 4C). These findings were further validated by an additional set of experiments in which we used the selective Sig-1R antagonist BD1063 to block Sig-1R-mediated signals (Figure S2). The effects of DMT on cellular survival and HIF-1α expression is thus dependent on the Sig-1R in human primary iPSC-derived neurons and monocyte-derived microglia-like cells.

## Discussion

In a previous study we reported the expression of Sig-1R in human moMACs and moDCs (Szabo et al., 2014). Although the expression of Sig-1R has been described in several human tissues its expression has not been investigated in iPSC-derived neurons. We therefore first examined the mRNA and protein level expression of Sig-1R in human *in vitro* differentiated iPSC-derived cortical neurons during the process of differentiation. We found, that NSCs have low baseline levels of Sig-1R mRNA and protein which are increasing during the differentiation process and both peaking at the end of differentiation (Figure 1). The increase in Sig-1R protein levels exhibited statistical significance from the 21st day of differentiation as compared to baseline (NSC) values (Figure 1B). It is in agreement with recent findings showing alterations in phenotypic similarities between primary tissue and iPSC-derived cortical neurons by the analysis of transcriptomic changes in several typical neural marker genes (Handel et al., 2016).

After demonstrating the expression of Sig-1R in our *in vitro* cultures we next sought to investigate the effects of DMT treatment in hypoxic stress. We applied a severe hypoxia model setting oxygen levels to 0.5% (94.5% N_2_, 5% CO_2_) in accordance with recent studies (Lee et al., 2013; Harrison et al., 2015). Hypoxia caused—dominantly apoptotic—cell death in all types of cultures within 6 h. Interestingly, DMT-treated cultures exhibited significantly higher survival rates as measured by flow cytometry. Relatively low *in vitro* working concentrations of DMT were found to significantly increase the survival of cells (10–50 μM for iPSC-derived cortical neurons, and 50 μM for moMACs/moDCs) as compared to non-treated controls (Figure 2). Cellular viability was less affected in hypoxia-exposed moMAC and moDC cultures (Figures 2B,C) in concert with recent findings reporting relative resistance to hypoxia of monocytes and monocyte-derived myeloid cells (Elbarghati et al., 2008).

To assess the influence of DMT on cellular physiology under hypoxia we monitored the expression and function of HIF-1α, an endogenous indicator of hypoxic stress (Semenza, 2002). We found that HIF-1α was rapidly upregulated in all cell types following 6 h of hypoxia exposure, a phenomenon that was significantly prevented by the administration of DMT (Figure 3). Consistent with these results, under severe hypoxia, mRNA expression of the HIF-1α target gene VEGF was also significantly lower in DMT-treated human primary cells as compared to control cultures (Figure 3C).

Taken together, our results suggested that DMT may prevent or mitigate cellular stress in hypoxic environments. This protective effect may be brought about through various mechanisms. Since one of the proposed mechanism is the DMT-mediated activation of Sig-1R (Frecska et al., 2013, 2016), we used the approach of gene-specific silencing in order to test the contribution of Sig-1R to the observed phenomenon. Our findings demonstrated that the downregulation of Sig-1R leads to the abrogation of DMT-mediated effects, as far as cellular survival and HIF-1α expressions are concerned, in hypoxic *in vitro* cultures of human primary cells (Figure 4). Furthermore, by using a Sig-1R-specific inhibitor we could further verify our results in all cell types in terms of cellular survival (Figure S2). These data suggest a critical, indispensable role of Sig-1R in the protective and antistress effects of DMT in human iPSC-derived cortical neurons, moMACs and moDCs in hypoxic environment.

The observed, Sig-1R-mediated protective and antistress effects of DMT in hypoxia may be based on multiple mechanisms. Since the Sig-1R has been shown to promote neuronal survival against oxidative stress (Pal et al., 2012) as well as to modulate immune processes (Szabo, 2015), it is tempting to speculate that its activation by DMT, a natural, endogenous ligand, also results in similar physiological phenomena. One of the possible mechanisms is the fine-tuning of mitochondrial functions and consequently the regulation of cellular oxygen metabolism, a process that is indirectly related to HIF-1α expression and function (Ramamoorthy and Shi, 2014). Secondly, Sig-1R activation by DMT may also result in modulated Ca^2+^ signaling altering the function of intracellular kinases involved in cellular survival (Bickler et al., 2004). Although altered mitochondrial functions may interfere with HIF-1α expression and activation, it has been shown that hypoxic stress can also be canalized downstream toward the nucleus through mitochondrion-associated intracellular stress pathways irrespective of HIF-1α (reviewed in Masson and Ratcliffe, 2014). Since Sig-1R is primarily residing in the MAM membrane system, its activation likely affects an HIF-1α-independent pathway. Our results support this hypothesis as DMT-treated human primary cells exhibited higher survival rates despite their decreased HIF-1α expression, a phenomenon that suggest either moderate hypoxic stress response or the activation of a HIF-1α-independent pathway (Figure 3). Although we could exclude some of these additional stress-related pathways, such as NF-κB and ATF6 (Figure S1), the biochemical elucidation of these background mechanisms needs further investigations.

This is the first study reporting that DMT, through the Sig-1R of human primary cells, can increase survival and alleviate cellular stress in hypoxic environments. This phenomenon is associated with increased cell viability and decreased expression and function of the stress factor HIF-1α in severe hypoxia-exposed, DMT-treated iPSC-derived cortical neurons, and monocyte-derived immune cells. The importance of microglia and microglia-like cells, such as monocytes, macrophages, and dendritic cells in hypoxia and post-injury recovery of the CNS has been recently reported (Jin and Yamashita, 2016). Thus, DMT may also notably contribute to neuroregenerative and neurorestorative processes by modulating the survival of microglia-like cells, such as moMACs and moDCs. In conclusion, our results suggest a novel and important role of DMT in human cellular physiology and point out to the relevance of DMT-mediated Sig-1R modulation in future therapies concerning hypoxia/ischemia-related pathologies.

## Author contributions

Conceived and designed the experiments: AS, AK, JR, SD, ER, EF. Performed the experiments: AS, AK, JR, EF. Analyzed the data: AS, JR, SD, EF. Contributed reagents/materials/analysis tools: ER, EF. Contributed to the writing of the manuscript: AS, AK, JR, SD, ER, EF.

### Conflict of interest statement

The authors declare that the research was conducted in the absence of any commercial or financial relationships that could be construed as a potential conflict of interest.

## Supplementary material

Figure S1 **DMT does not influence the hypoxia-modulated expression of the ER-stress sensor ATF6 in human primary cells. (A)** Prior to their placement into hypoxia chamber (0.5% O_2_), cells were treated with 50 μM DMT (+DMT) or left non-treated (ctrl). Protein level expression of ATF6 was evaluated by Western blotting after 6 h of hypoxia treatment (6 h) in comparison with the baseline expression (0 h). A control experiment was performed at normal oxygen level (normoxia). Results of a typical experiment out of three is shown. **(B)** Densitometry analysis of ATF6 Western blot data in hypoxia using the same experimental setup as in Figure 3 and panel **(A)**. Relative density values are presented as Mean ± SEM of three independent hypoxia experiments. Neuron, human iPSC-derived cortical neuron; moMAC, human monocyte-derived macrophage; moDC, human monocyte-derived dendritic cell.

Figure S2 **The effect of Sig-1R inhibition on the DMT-mediated cell survival of human iPSC-derived cortical neurons, moMACs and moDCs in hypoxia**. Specific Sig-1R blocking was performed by using the highly selective Sig-1R antagonist BD1063 dihydrochloride at working concentrations of 1–100 μM. Cellular survival was monitored similarly to Figures 2, 4. Cells were treated with 50 μM DMT before hypoxia treatment (hypoxia+DMT) or left untreated (hypoxia ctrl). Cultures with 30 min prior exposure to the antagonist (hypoxia+DMT+1–100 μM BD1063) were also tested within the same experimental setup. Results of three independent experiments are shown as Mean ± SEM. Asterisk indicates statistical significance as compared to hypoxia+DMT (*p* < 0.05).

## References

[B1] Axelrod J. (1961). Enzymatic formation of psychotomimetic metabolites from normally occurring compounds. Science 134:343. 10.1126/science.134.3475.343 13685339

[B2] Barker S. A. Monti J. A. Christian S. T. (1981). N, N-dimethyltryptamine: an endogenous hallucinogen. Int. Rev. Neurobiol. 22, 83–110. 10.1016/S0074-7742(08)60291-3 6792104

[B3] Baulieu E. E. (1998). Neurosteroids: a novel function of the brain. Psychoneuro endocrinology 23, 963–987. 10.1016/S0306-4530(98)00071-7 9924747

[B4] Beaton J. M. Morris P. E. (1984). Ontogeny of N,N-dimethyltryptamine and related indolealkylamine levels in neonatal rats. Mech. Ageing Dev. 25, 343–347. 10.1016/0047-6374(84)90007-1 6588281

[B5] Bickler P. E. Fahlman C. S. Ferriero D. M. (2004). Hypoxia increases calcium flux through cortical neuron glutamate receptors via protein kinase C. J. Neurochem. 88, 878–884. 10.1046/j.1471-4159.2003.02203.x 14756808

[B6] Brahimi-Horn M. C. Pouysségur J. (2007). Oxygen, a source of life and stress. FEBS Lett. 581, 3582–3591. 10.1016/j.febslet.2007.06.018 17586500

[B7] Cheong C. Matos I. Choi J. H. Dandamudi D. B. Shrestha E. Longhi M. P.. (2010). Microbial stimulation fully differentiates monocytes to DC-SIGN/CD209(+) dendritic cells for immune T cell areas. Cell 143, 416–429. 10.1016/j.cell.2010.09.039 21029863 PMC3150728

[B8] Cuevas J. Behensky A. Deng W. Katnik C. (2011a). Afobazole modulates neuronal response to ischemia and acidosis via activation of sigma-1 receptors. J. Pharmacol. Exp. Ther. 339, 152–160. 10.1124/jpet.111.182774 21715562

[B9] Cuevas J. Rodriguez A. Behensky A. Katnik C. (2011b). Afobazole modulates microglial function via activation of both sigma-1 and sigma-2 receptors. J. Pharmacol. Exp. Ther. 339, 161–172. 10.1124/jpet.111.182816 21715561

[B10] Elbarghati L. Murdoch C. Lewis C. E. (2008). Effects of hypoxia on transcription factor expression in human monocytes and macrophages. Immunobiology 213, 899–908. 10.1016/j.imbio.2008.07.016 18926304

[B11] Fontanilla D. Johannessen M. Hajipour A. R. Cozzi N. V. Jackson M. B. Ruoho A. E. (2009). The hallucinogen N,N-dimethyltryptamine (DMT) is an endogenous sigma-1 receptor regulator. Science 323, 934–937. 10.1126/science.1166127 19213917 PMC2947205

[B12] Franzen F. Gross H. (1965). Tryptamine, N,N-dimethyltryptamine, N,N-dimethyl-5-hydroxytryptamine and 5-methoxytryptamine in human blood and urine. Nature 206, 1052. 10.1038/2061052a0 5839067

[B13] Frecska E. Bokor P. Winkelman M. (2016). The therapeutic potentials of ayahuasca: possible effects against various diseases of civilization. Front. Pharmacol. 7:35. 10.3389/fphar.2016.00035 26973523 PMC4773875

[B14] Frecska E. Szabo A. Winkelman M. J. Luna L. E. McKenna D. J. (2013). A possibly sigma-1 receptor mediated role of dimethyltryptamine in tissue protection, regeneration, and immunity. J. Neural. Transm. (Vienna) 120, 1295–1303. 10.1007/s00702-013-1024-y 23619992

[B15] Handel A. E. Chintawar S. Lalic T. Whiteley E. Vowles J. Giustacchini A.. (2016). Assessing similarity to primary tissue and cortical layer identity in induced pluripotent stem cell-derived cortical neurons through single-cell transcriptomics. Hum. Mol. Genet. 25, 989–1000. 10.1093/hmg/ddv637 26740550 PMC4754051

[B16] Harrison D. K. Fasching M. Fontana-Ayoub M. Gnaiger E. (2015). Cytochrome redox states and respiratory control in mouse and beef heart mitochondria at steady-state levels of hypoxia. J. Appl. Physiol. (1985) 119, 1210–1218. 10.1152/japplphysiol.00146.2015 26251509

[B17] Hayashi T. Su T. P. (2007). Sigma-1 receptor chaperones at the ER-mitochondrion interface regulate Ca(2+) signaling and cell survival. Cell 131, 596–610. 10.1016/j.cell.2007.08.036 17981125

[B18] Heilker R. Traub S. Reinhardt P. Schöler H. R. Sterneckert J. (2014). iPS cell derived neuronal cells for drug discovery. Trends Pharmacol. Sci. 35, 510–519. 10.1016/j.tips.2014.07.003 25096281

[B19] Hellewell S. B. Bruce A. Feinstein G. Orringer J. Williams W. Bowen W. D. (1994). Rat liver and kidney contain high densities of sigma 1 and sigma 2 receptors: characterization by ligand binding and photoaffinity labeling. Eur. J. Pharmacol. 268, 9–18. 10.1016/0922-4106(94)90115-5 7925616

[B20] Huang L. E. Gu J. Schau M. Bunn H. F. (1998). Regulation of hypoxia-inducible factor 1alpha is mediated by an O_2_-dependent degradation domain via the ubiquitin-proteasome pathway. Proc. Natl. Acad. Sci. U.S.A. 95, 7987–7992. 10.1073/pnas.95.14.7987 9653127 PMC20916

[B21] Jin X. Yamashita T. (2016). Microglia in central nervous system repair after injury. J. Biochem. 159, 491–496. 10.1093/jb/mvw009 26861995

[B22] Kallio P. J. Wilson W. J. O'Brien S. Makino Y. Poellinger L. (1999). Regulation of the hypoxia-inducible transcription factor 1alpha by the ubiquitin-proteasome pathway. J. Biol. Chem. 274, 6519–6525. 10.1074/jbc.274.10.6519 10037745

[B23] Katnik C. Guerrero W. R. Pennypacker K. R. Herrera Y. Cuevas J. (2006). Sigma-1 receptor activation prevents intracellular calcium dysregulation in cortical neurons during *in vitro* ischemia. J. Pharmacol. Exp. Ther. 319, 1355–1365. 10.1124/jpet.106.107557 16988055

[B24] Klouz A. Saïd D. B. Ferchichi H. Kourda N. Ouanes L. Lakhal M.. (2008). Protection of cellular and mitochondrial functions against liver ischemia by N-benzyl-N′-(2-hydroxy-3,4-dimethoxybenzyl)-piperazine (BHDP), a sigma1 ligand. Eur. J. Pharmacol. 578, 292–299. 10.1016/j.ejphar.2007.09.038 17964567

[B25] Lee H. Y. Yang E. G. Park H. (2013). Hypoxia enhances the expression of prostate-specific antigen by modifying the quantity and catalytic activity of Jumonji C domain-containing histone demethylases. Carcinogenesis 34, 2706–2715. 10.1093/carcin/bgt256 23884959

[B26] Liu L. Simon M. C. (2004). Regulation of transcription and translation by hypoxia. Cancer Biol. Ther. 3, 492–497. 10.4161/cbt.3.6.1010 15254394

[B27] London A. Cohen M. Schwartz M. (2013). Microglia and monocyte-derived macrophages: functionally distinct populations that act in concert in CNS plasticity and repair. Front. Cell. Neurosci. 7:34. 10.3389/fncel.2013.00034 23596391 PMC3625831

[B28] Mancuso R. Olivan S. Rando A. Casas C. Osta R. Navarro X. (2012). Sigma-1R agonist improves motor function and motoneuron survival in ALS mice. Neurotherapeutics 9, 814–826. 10.1007/s13311-012-0140-y 22935988 PMC3480575

[B29] Masson N. Ratcliffe P. J. (2014). Hypoxia signaling pathways in cancer metabolism: the importance of co-selecting interconnected physiological pathways. Cancer Metab. 2:3. 10.1186/2049-3002-2-3 24491179 PMC3938304

[B30] Maurice T. Su T. P. (2009). The pharmacology of sigma-1 receptors. Pharmacol. Ther. 124, 195–206. 10.1016/j.pharmthera.2009.07.001 19619582 PMC2785038

[B31] McKenna D. Riba J. (2015). New world tryptamine hallucinogens and the neuroscience of ayahuasca. Curr. Top. Behav. Neurosci. [Epub ahead of print]. 10.1007/7854_2015_368. 25655746

[B32] Mohyeldin A. Garzon-Muvdi T. Quiñones-Hinojosa A. (2010). Oxygen in stem cell biology: a critical component of the stem cell niche. Cell Stem Cell 7, 150–161. 10.1016/j.stem.2010.07.007 20682444

[B33] Mori T. Hayashi T. Hayashi E. Su T. P. (2013). Sigma-1 receptor chaperone at the ER-mitochondrion interface mediates the mitochondrion-ER-nucleus signaling for cellular survival. PLoS ONE 8:e76941. 10.1371/journal.pone.0076941 24204710 PMC3799859

[B34] Pal A. Fontanilla D. Gopalakrishnan A. Chae Y. K. Markley J. L. Ruoho A. E. (2012). The sigma-1 receptor protects against cellular oxidative stress and activates antioxidant response elements. Eur. J. Pharmacol. 682, 12–20. 10.1016/j.ejphar.2012.01.030 22381068 PMC3314091

[B35] Penas C. Pascual-Font A. Mancuso R. Fores J. Casas C. Navarro X. (2011). Sigma receptor agonist 2-(4-morpholinethyl)1 phenylcyclohexanecarboxylate (Pre084) increases GDNF and BiP expression and promotes neuroprotection after root avulsion injury. J. Neurotrauma 28, 831–840. 10.1089/neu.2010.1674 21332255

[B36] Ramamoorthy P. Shi H. (2014). Ischemia induces different levels of hypoxia inducible factor-1alpha protein expression in interneurons and pyramidal neurons. Acta Neuropathol. Commun. 2:51. 10.1186/2051-5960-2-51 24887017 PMC4035094

[B37] Ruscher K. Inacio A. R. Valind K. Rowshan Ravan A. Kuric E. Wieloch T. (2012). Effects of the sigma-1 receptor agonist 1-(3,4-dimethoxyphenethyl)-4-(3-phenylpropyl)-piperazine dihydro-chloride on inflammation after stroke. PLoS ONE 7:e45118. 10.1371/journal.pone.0045118 23028794 PMC3445585

[B38] Saavedra J. M. Axelrod J. (1972). Psychotomimetic N-methylated tryptamines: formation in brain *in vivo* and *in vitro*. Science 175, 1365–1366. 5059565 10.1126/science.175.4028.1365

[B39] Saito T. Hanai S. Takashima S. Nakagawa E. Okazaki S. Inoue T.. (2011). Neocortical layer formation of human developing brains and lissencephalies: consideration of layer-specific marker expression. Cereb. Cortex 21, 588–596. 10.1093/cercor/bhq125 20624841

[B40] Sakakibara T. Sukigara S. Saito T. Otsuki T. Takahashi A. Kaneko Y.. (2012). Delayed maturation and differentiation of neurons in focal cortical dysplasia with the transmantle sign: analysis of layer-specific marker expression. J. Neuropathol. Exp. Neurol. 71, 741–749. 10.1097/NEN.0b013e318262e41a 22805777

[B41] Semenza G. (2002). Signal transduction to hypoxia-inducible factor 1. Biochem. Pharmacol. 64, 993–998. 10.1016/S0006-2952(02)01168-1 12213597

[B42] Semenza G. L. (1999). Regulation of mammalian O_2_ homeostasis by hypoxia-inducible factor 1. Annu. Rev. Cell Dev. Biol. 15, 551–578. 10.1146/annurev.cellbio.15.1.551 10611972

[B43] Shen H. W. Jiang X. L. Winter J. C. Yu A. M. (2010). Psychedelic 5-methoxy-N,N-dimethyltryptamine: metabolism, pharmacokinetics, drug interactions, and pharmacological actions. Curr. Drug Metab. 11, 659–666. 10.2174/138920010794233495 20942780 PMC3028383

[B44] Shen H. W. Jiang X. L. Yu A. M. (2011). Nonlinear pharmacokinetics of 5-methoxy-N,N-dimethyltryptamine in mice. Drug Metab. Dispos. 39, 1227–1234. 10.1124/dmd.111.039107 21464174 PMC3127237

[B45] Simon M. C. Keith B. (2008). The role of oxygen availability in embryonic development and stem cell function. Nat. Rev. Mol. Cell Biol. 9, 285–296. 10.1038/nrm2354 18285802 PMC2876333

[B46] Steinman R. M. Banchereau J. (2007). Taking dendritic cells into medicine. Nature 449, 419–426. 10.1038/nature06175 17898760

[B47] Strassman R. J. Qualls C. R. (1994). Dose-response study of N,N-dimethyltryptamine in humans. I. Neuroendocrine, autonomic, and cardiovascular effects. Arch. Gen. Psychiatry 51, 85–97. 8297216 10.1001/archpsyc.1994.03950020009001

[B48] Su T. P. (1982). Evidence for sigma opioid receptor: binding of [3H]SKF-10047 to etorphine-inaccessible sites in guinea-pig brain. J. Pharmacol. Exp. Ther. 223, 284–290. 6290634

[B49] Su T. P. Hayashi T. Vaupel D. B. (2009). When the endogenous hallucinogenic trace amine N,N-dimethyltryptamine meets the sigma-1 receptor. Sci. Signal 2:pe12. 10.1126/scisignal.261pe12 19278957 PMC3155724

[B50] Su T. P. London E. D. Jaffe J. H. (1988). Steroid binding at sigma receptors suggests a link between endocrine, nervous, and immune systems. Science 240, 219–221. 2832949 10.1126/science.2832949

[B51] Su T.-P. Su T.-C. Nakamura Y. Tsai S.-Y. (2016). The Sigma-1 receptor as a pluripotent modulator in living systems. Trends Pharmacol. Sci. 37, 262–278. 10.1016/j.tips.2016.01.003 26869505 PMC4811735

[B52] Szabo A. (2015). Psychedelics and immunomodulation: novel approaches and therapeutic opportunities. Front. Immunol. 6:358. 10.3389/fimmu.2015.00358 26236313 PMC4500993

[B53] Szabo A. Fekete T. Koncz G. Kumar B. V. Pazmandi K. Foldvari Z.. (2016). RIG-I inhibits the MAPK-dependent proliferation of BRAF mutant melanoma cells via MKP-1. Cell. Signal. 28, 335–347. 10.1016/j.cellsig.2016.01.012 26829212

[B54] Szabo A. Kovacs A. Frecska E. Rajnavolgyi E. (2014). Psychedelic N,N-dimethyltryptamine and 5-methoxy-N,N-dimethyltryptamine modulate innate and adaptive inflammatory responses through the sigma-1 receptor of human monocyte-derived dendritic cells. PLoS ONE 9:e106533. 10.1371/journal.pone.0106533 25171370 PMC4149582

[B55] Szabo A. Rajnavolgyi E. (2013). Collaboration of Toll-like and RIG-I-like receptors in human dendritic cells: tRIGgering antiviral innate immune responses. Am. J. Clin. Exp. Immunol. 2, 195–207. 24179728 PMC3808934

[B56] Theodorou V. Chovet M. Eutamene H. Fargeau H. Dassaud M. Toulouse M.. (2002). Antidiarrhoeal properties of a novel sigma ligand (JO 2871) on toxigenic diarrhoea in mice: mechanisms of action. Gut 51, 522–528. 10.1136/gut.51.4.522 12235074 PMC1773374

[B57] Wohleb E. S. Powell N. D. Godbout J. P. Sheridan J. F. (2013). Stress-induced recruitment of bone marrow-derived monocytes to the brain promotes anxiety-like behavior. J. Neurosci. 33, 13820–13833. 10.1523/JNEUROSCI.1671-13.2013 23966702 PMC3755721

[B58] Wolfe S. A. Jr. Kulsakdinun C. Battaglia G. Jaffe J. H. De Souza E. B. (1988). Initial identification and characterization of sigma receptors on human peripheral blood leukocytes. J. Pharmacol. Exp. Ther. 247, 1114–1119. 2849660

[B59] Wu D. Yotnda P. (2011). Induction and testing of hypoxia in cell culture. J. Vis. Exp. pii:e2899. 10.3791/2899 21860378 PMC3217626

