# Supplementary figures and images for: The Endogenous Hallucinogen and Trace Amine N,N-Dimethyltryptamine (DMT) Displays Potent Protective Effects against Hypoxia via Sigma-1 Receptor Activation in Human Primary iPSC-Derived Cortical Neurons and Microglia-Like Immune Cells
Source: Front Neurosci. 2016 Sep 14;10:423. doi: 10.3389/fnins.2016.00423 (PMC5021697; doi:10.3389/fnins.2016.00423)

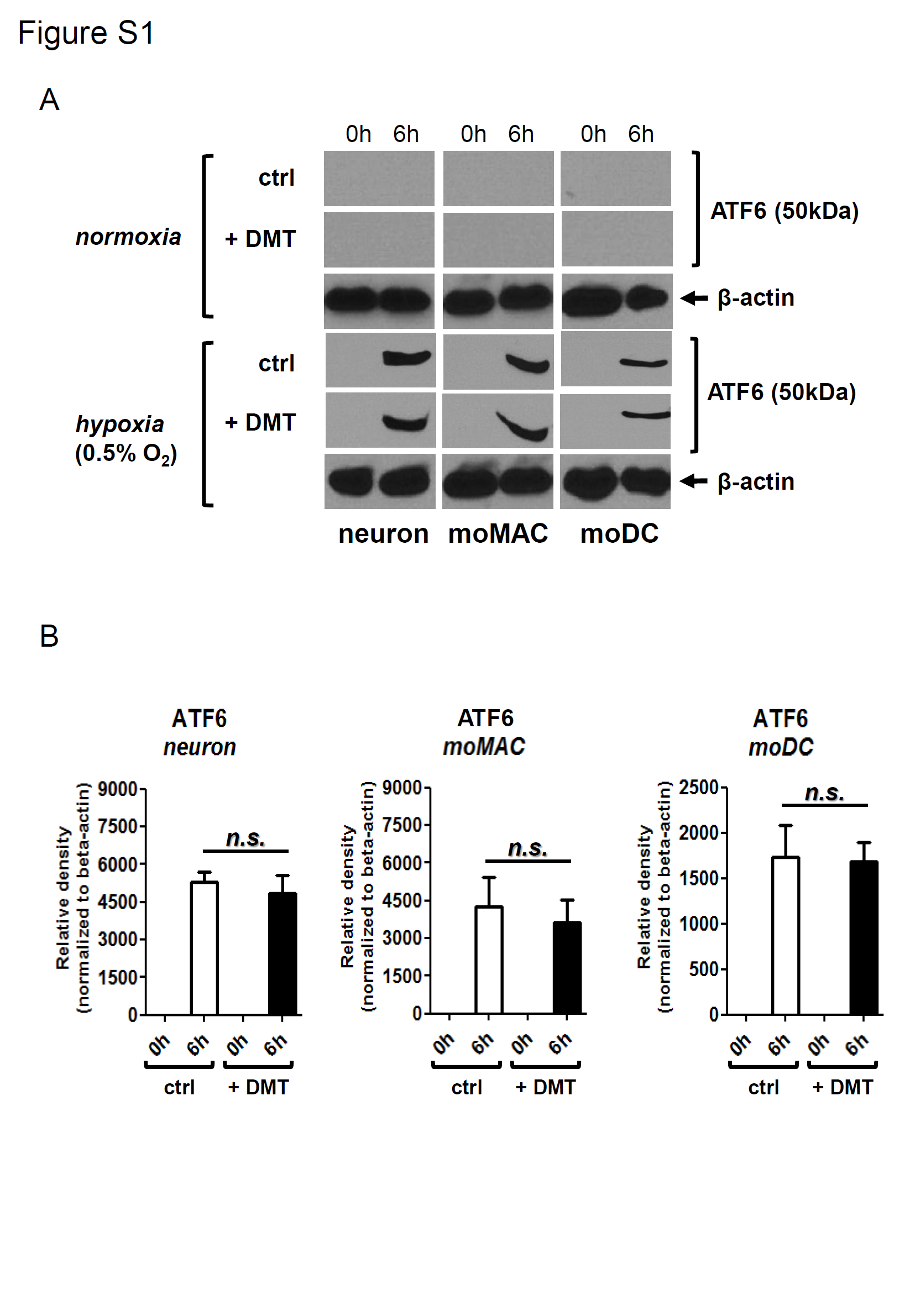

Supplement: Figure S1 — DMT does not influence the hypoxia-modulated expression of the ER-stress sensor ATF6 in human primary cells. (A) Prior to their placement into hypoxia chamber (0.5% O2), cells were treated with 50 μM DMT (+DMT) or left non-treated (ctrl). Protein level expression of ATF6 was evaluated by Western blotting after 6 h of hypoxia treatment (6 h) in comparison with the baseline expression (0 h). A control experiment was performed at normal oxygen level (normoxia). Results of a typical experiment out of three is shown. (B) Densitometry analysis of ATF6 Western blot data in hypoxia using the same experimental setup as in Figure 3 and panel (A). Relative density values are presented as Mean ± SEM of three independent hypoxia experiments. Neuron, human iPSC-derived cortical neuron; moMAC, human monocyte-derived macrophage; moDC, human monocyte-derived dendritic cell. [file Image1.TIF]

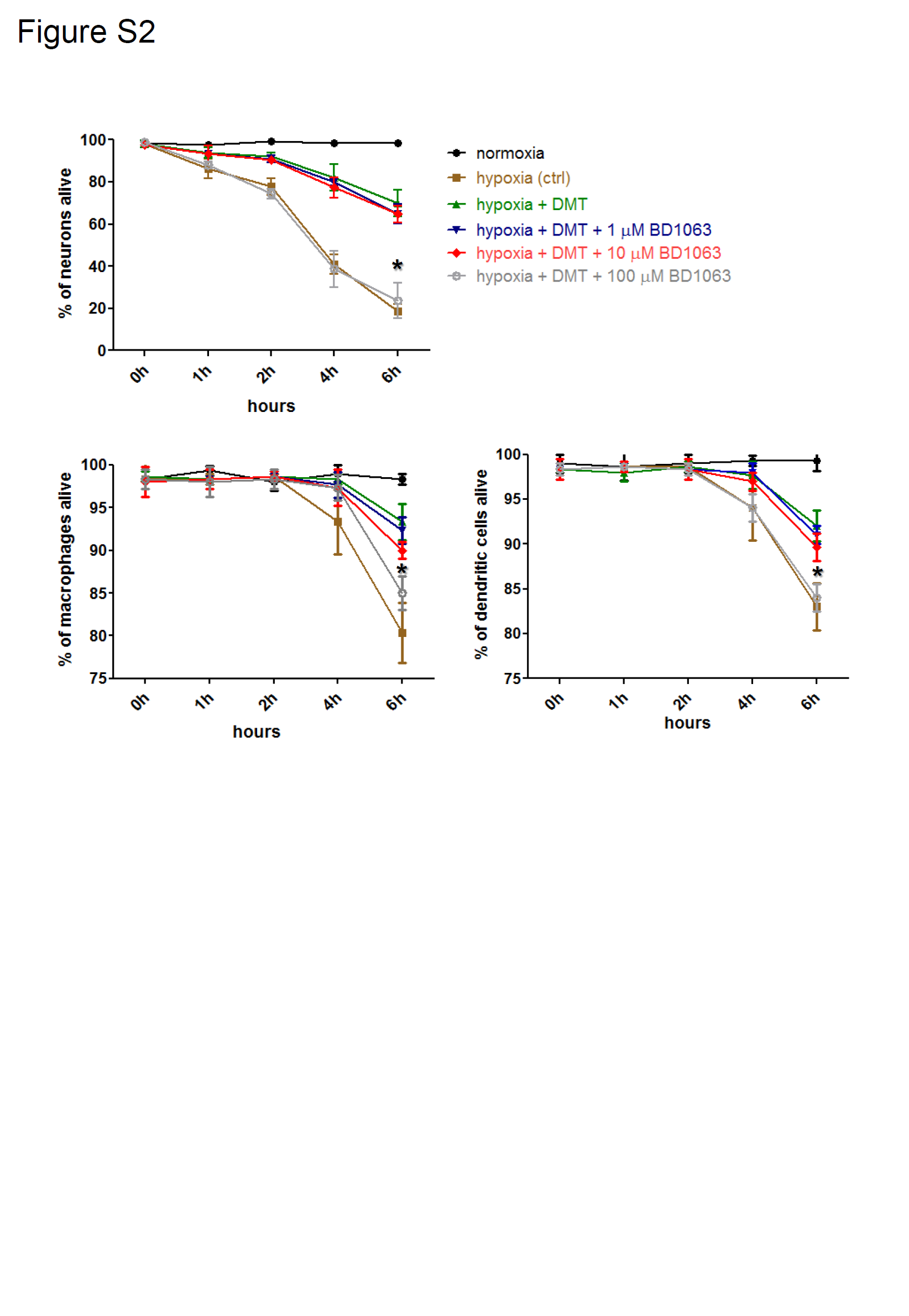

Supplement: Figure S2 — The effect of Sig-1R inhibition on the DMT-mediated cell survival of human iPSC-derived cortical neurons, moMACs and moDCs in hypoxia. Specific Sig-1R blocking was performed by using the highly selective Sig-1R antagonist BD1063 dihydrochloride at working concentrations of 1–100 μM. Cellular survival was monitored similarly to Figures 2, 4. Cells were treated with 50 μM DMT before hypoxia treatment (hypoxia+DMT) or left untreated (hypoxia ctrl). Cultures with 30 min prior exposure to the antagonist (hypoxia+DMT+1–100 μM BD1063) were also tested within the same experimental setup. Results of three independent experiments are shown as Mean ± SEM. Asterisk indicates statistical significance as compared to hypoxia+DMT (p < 0.05). [file Image2.TIF]
